# Small Extracellular Vesicles Isolated from Serum May Serve as Signal-Enhancers for the Monitoring of CNS Tumors

**DOI:** 10.3390/ijms21155359

**Published:** 2020-07-28

**Authors:** Gabriella Dobra, Matyas Bukva, Zoltan Szabo, Bella Bruszel, Maria Harmati, Edina Gyukity-Sebestyen, Adrienn Jenei, Monika Szucs, Peter Horvath, Tamas Biro, Almos Klekner, Krisztina Buzas

**Affiliations:** 1 Laboratory of Microscopic Image Analysis and Machine Learning, Institute of Biochemistry, Biological Research Centre, H-6726 Szeged, Hungary; dobragab@yahoo.co.uk (G.D.); bukvamatyas@gmail.com (M.B.); harmatimarcsi@gmail.com (M.H.); e.gyukity.sebestyen@gmail.com (E.G.-S.); horvath.peter@brc.hu (P.H.); 2 Department of Medical Genetics, Doctoral School of Interdisciplinary Medicine, University of Szeged, H-6720 Szeged, Hungary; 3 Department of Medical Chemistry, Faculty of Medicine, University of Szeged, H-6720 Szeged, Hungary; szabo.zoltan@med.u-szeged.hu (Z.S.); bruszel.bella@med.u-szeged.hu (B.B.); 4 Department of Neurosurgery, Clinical Centre, University of Debrecen, H-4032 Debrecen, Hungary; jenei.adrienn@med.unideb.hu (A.J.); aklekner@yahoo.com (A.K.); 5 Department of Medical Physics and Informatics, Faculty of Medicine, University of Szeged, H-6720 Szeged, Hungary; szucs.monika@med.u-szeged.hu; 6 Department of Medical Physics and Informatics, Faculty of Science and Informatics, University of Szeged, H-6720 Szeged, Hungary; 7 Department of Immunology, Faculty of Medicine, University of Debrecen, H-4032 Debrecen, Hungary; biro.lcmp@gmail.com; 8 Department of Immunology, Faculty of Medicine, University of Szeged, H-6720 Szeged, Hungary; 9 Department of Immunology, Faculty of Science and Informatics, University of Szeged, H-6720 Szeged, Hungary

**Keywords:** extracellular vesicles, cancer biomarker, proteomics

## Abstract

Liquid biopsy-based methods to test biomarkers (e.g., serum proteins and extracellular vesicles) may help to monitor brain tumors. In this proteomics-based study, we aimed to identify a characteristic protein fingerprint associated with central nervous system (CNS) tumors. Overall, 96 human serum samples were obtained from four patient groups, namely glioblastoma multiforme (GBM), non-small-cell lung cancer brain metastasis (BM), meningioma (M) and lumbar disc hernia patients (CTRL). After the isolation and characterization of small extracellular vesicles (sEVs) by nanoparticle tracking analysis (NTA) and atomic force microscopy (AFM), liquid chromatography -mass spectrometry (LC-MS) was performed on two different sample types (whole serum and serum sEVs). Statistical analyses (ratio, Cohen’s d, receiver operating characteristic; ROC) were carried out to compare patient groups. To recognize differences between the two sample types, pairwise comparisons (Welch’s test) and ingenuity pathway analysis (IPA) were performed. According to our knowledge, this is the first study that compares the proteome of whole serum and serum-derived sEVs. From the 311 proteins identified, 10 whole serum proteins and 17 sEV proteins showed the highest intergroup differences. Sixty-five proteins were significantly enriched in sEV samples, while 129 proteins were significantly depleted compared to whole serum. Based on principal component analysis (PCA) analyses, sEVs are more suitable to discriminate between the patient groups. Our results support that sEVs have greater potential to monitor CNS tumors, than whole serum.

## 1. Introduction

According to the World Health Organization (WHO), cancer is the second leading cause of death, accounting for an estimated 9.6 million cases in 2018. Globally, 1 in 6 deaths is due to cancer [1]. The cancer burden continues to grow worldwide, exerting tremendous physical, emotional and financial strain on individuals, families, communities and on health systems [2].

The diagnosis of central nervous system (CNS) tumors is based on CT and MRI scans, as well as on the histopathological analysis of samples obtained by biopsy or via surgical resection. However, these procedures are highly invasive, uncomfortable for the patient, bear a considerable risk of complications and provide limited information on tumor status. Therefore, biomarkers appropriate for monitoring disease progression and response to treatment are eagerly required. While repeated MRI scans serve as the standard method to follow patients, it has little prognostic value for long-term recurrence [3]. Thus, neuro-oncological research aims to identify novel biomarkers suitable for monitoring CNS tumors in clinical practice [4].

Liquid biopsy is in the spotlight of biomarker-focused research, as body fluids are easily accessible sources of biomarkers and are available with minimally invasive and low cost sampling procedures. Also, multiple sampling allows the monitoring of disease progression and therapeutic response [5]. Every cell, including neoplastic cells, release molecular markers into the circulation. Tumor-derived biomarkers include proteins, nucleic acids, circulating tumor cells, platelets and tumor-derived extracellular vesicles that accumulate in urine, cerebrospinal fluid, saliva and blood [6].

Blood is the most easily accessible source for biomarkers, thus it is frequently used to assess disease status in malignancies such as prostate, liver and ovarian cancers based on the serum concentrations of PSA, AFP and CA125, respectively. In neuro-oncology, blood-based biomarkers are mainly used to evaluate toxicity and safety of treatments to guide patient management. For example, myelosuppression is a common risk associated with temozolomide treatment and radiotherapy, thus standard practice dictates weekly tests of complete blood count, including whole blood cell differential and platelet counts during definitive chemoradiotherapy [7]. Finding biomarkers for blood-based CNS tumor monitoring is more challenging, as the blood-brain barrier (BBB) prevents the release of tumor-related biomarkers into peripheral blood. However, it would have outstanding benefits in clinical patient management, thus efforts to identify blood based biomarkers, including proteins, nucleic acids, circulating tumor cells and extracellular vesicles are currently in the forefront of neuro-oncological research [8].

Extracellular vesicles (EVs) are promising cancer biomarkers accessible via liquid biopsy, because they are cell-secreted, nano-sized and stably exist in all types of body fluids. EVs contain a sample of the cytosolic milieu, including an abundance of DNA, RNA, proteins and other analytes, while externally they also resemble their cell of origin [9]. EVs are small, lipid bilayer-enclosed vesicles released by both cancer and non-cancerous cells into the extracellular space [10].

EVs secreted by cancer cells communicate with neighboring stromal cells or even with cells at distant sites, inducing an alteration of the cell program [11,12]. Pre-metastatic niche formation has been shown in several tumors, for example, in pancreatic, lung, colorectal and ovarian cancers [13,14,15,16]. Also, EVs may be taken up by immune cells, leading to immunosuppression [17]. More recently, EVs have even gained a role in cancer diagnosis and therapy [18,19,20] as biomarker molecules that may be identified in different primary tumors with high sensitivity and specificity [21]. Regarding pancreatic cancer, Kalluri and colleagues found that glypican-1 (GPC1), a cell surface proteoglycan, is specifically enriched in circulating exosomes (30–200 nm endosome-derived EVs). GPC1 is suitable to differentiate early- and late-stage pancreatic cancer from benign diseases of the pancreas, with an accuracy of 100% [22]. The available evidence also supports that tumor-derived EVs can cross the BBB [23,24], however, currently no clinically relevant EV biomarkers are accepted for the monitoring of CNS tumors.

Several studies report on gene or protein expression analyses of CNS tumor tissue (specifically, glioblastoma), allowing to identify biomarkers that could be secreted into the blood and thus could be detected from serum samples. Recent studies have aimed to identify one and two specific biomarkers for the reliable evaluation of actual tumor status [25,26,27] but none of these proteins alone was found to be sufficiently specific and sensitive to serve as a monitoring marker.

Regarding that previous attempts to find surrogate serum markers for brain tumors have failed when based on a single or only few candidate factors, we made an attempt to identify a characteristic protein fingerprint of 10–20 candidate markers associated with CNS tumors.

For this purpose, 96 serum samples were collected from four patient groups according to the criteria of the National Ethical Committee and proteomics analysis was performed using liquid chromatography and mass spectrometry (LC-MS). The serum samples were obtained from patients diagnosed with the two most common types of brain tumors [28], namely malignant glioblastoma multiforme (GBM) and benign meningioma (M), as well as from patients with a prevalent brain metastasis [29] originating from non-small-cell lung cancer (BM). Patients with lumbar disc herniation served as controls (CTRL). Following a statistical selection, these four patient groups were compared with respect to the identified proteins. In parallel, small extracellular vesicles (sEVs) were isolated from the serum samples by differential centrifugation and proteomics and statistical analyses were also performed on these sEV samples, allowing to compare the suitability of these two different sample types. According to the best of our knowledge, this is the first study that compares the proteome of whole serum and serum-derived sEV samples. Results from the proteomics analysis indicate that using a protein fingerprint of serum-derived sEVs instead of analyzing whole serum increases the accuracy of distinguishing between the clinical samples, that is, between the patient groups. Our results support that sEVs have a greater potential for the proteomics-based monitoring of CNS tumors compared to whole serum analysis.

## 2. Results

### 2.1. EV Samples Show sEV Properties with Similar Concentration and Size Distribution in the Different Patient Groups

To verify the value of circulating extracellular vesicles as potential biomarkers for CNS tumors, EVs were isolated from the serum of patients with glioblastoma multiforme (GBM), single brain metastasis originating from non-small-cell lung cancer (BM) and meningioma (M), as well as from control patients with lumbar disc herniation (CTRL). Each group included 24 individuals of both genders with various ages. Extracellular vesicles were isolated from the sera by differential centrifugation and were characterized by atomic force microscopy (AFM) and nanoparticle tracking analysis (NTA). Pools of 6 samples were formed in all groups, allowing four parallel samples to be tested per group (see in Section 4.1). Western blot analyses were also performed to demonstrate the EV nature (Figure S1).

**Figure 1:**
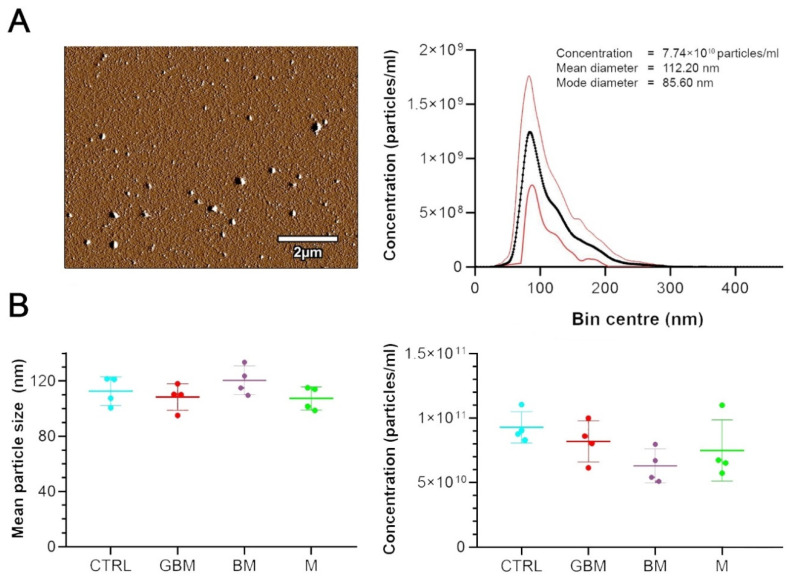
Characterization and quantitative properties of the small extracellular vesicle (sEV) samples. (**A**) Atomic force microscopy (AFM) image of sEV isolates displays vesicles with diameters within the range of 50–140 nm. The diagram shows the size distribution of the 96 sEV samples isolated from the serum, presenting the mean +/−95% CI values measured by nanoparticle tracking analysis (NTA). (**B**) Dot plots show the number and size distribution of small extracellular vesicles (sEVs) displayed in mean size (left) and concentration (right) values for each sample pools (4 samples/group).

EVs were divided into subtypes based on their size range, separating small EVs (sEVs) and medium/large EVs (m/lEVs) [30]. AFM analysis revealed that the small EV subtype includes various structures. Mean and mode diameters of the particles, represented by an average of the 16 sample pools, were measured as 112 nm and 86 nm by NTA, respectively (Figure 1A).

The quantitative characterization of serum sEVs by NTA (Figure 1B) revealed no significant differences between the four patient groups regarding the size and concentration of circulating sEVs. However, within the groups high individual differences were observed in the measured parameters of the sEVs.

### 2.2. Statistical Analysis of LC-MS Data Reveals Characteristic Proteomic Fingerprints for Each Patient Group and Informs on the Suitability of the Two Different Sample Types in Distinguishing CNS Tumors

**Figure 2:**
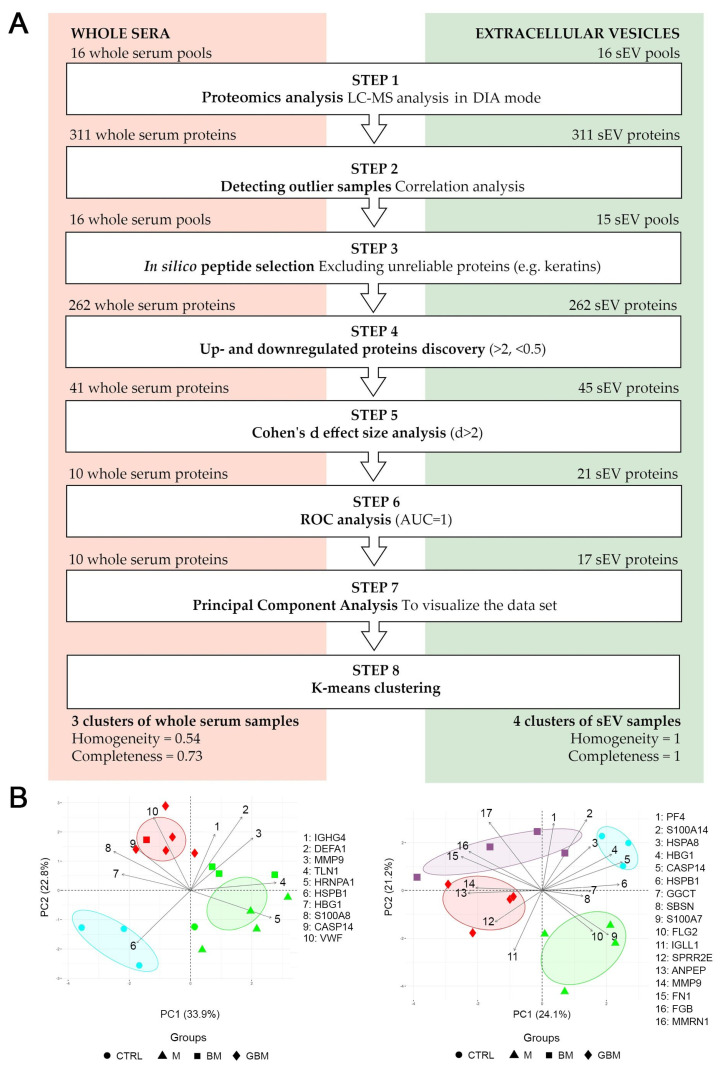
Statistical analysis of the proteome of whole serum (left) and sEV samples (right). (**A**) The flowchart shows the steps of selecting the proteins revealed by liquid chromatography and mass spectrometry (LC-MS) (**B**) The diagrams visualize the results of the principal component analysis (PCA) and k-means clustering. X and Y axes of PCA biplots show principal component 1 (PC1) and principal component 2 (PC2) with explained variances. Arrows represent the coefficients of each protein for PC1 versus the coefficients for PC2, showing the significance of each protein in influencing PCs. Different dots represent the 4 patient groups. Colors indicate the clusters formed by k-means clustering; ellipses indicating the 95% confidence interval were constructed around the barycenters of the clusters.

We aimed to identify the differences between the four patient groups to reveal the characteristic protein profiles associated with the CNS tumors in point. Using an intensity ratio of >2 or <0.5 with Cohen’s d effect size of 2 as a cut-off, we investigated which proteins show reliable intensity difference and which proteins can separate at least one group from the others based on a receiver operating characteristic (ROC) analysis. Moreover, utilizing principal component analysis (PCA) with k-means clustering, we were able to compare the suitability of the two different sample types to distinguish between the CNS tumors in point. Figure 2 shows the flowchart of LC-MS data processing and the results of the statistical analyses.

Proteomics analyses by LC-MS (Step 1) were performed on whole serum and sEV samples obtained from patients with GBM, BM, M and CTRL. Individual samples (*n* = 24) in each group were arranged into 4 pools (see in Section 4.1) to eliminate individual variances, reduce sample number, shorten the time of LC-MS measurements and reduce the need for materials. The Data independent acquisition (DIA) mode constructed spectral library revealed 311 proteins (see Table S1). Based on Pearson’s correlation analyses (Step 2), one of the sEV control samples had to be excluded from further statistical analyses (Table S2). After excluding unreliable proteins, as well as proteins with missing values (Step 3), a total of 262 proteins remained for the final analysis.

Following basic processing, up- and down-regulated protein discovery (Step 4) resulted in 41 whole serum proteins and 45 sEV proteins. In addition to comparing each CNS tumor group to CTRL, between-group differences among the CNS tumor groups were also assessed in the protein selection process. As clinically relevant incidence is an important consideration for selecting the proteins identified, Cohen’s d effect size was adopted as an indicator of between-groups difference. The Cohen’s d effect size analysis (Step 5) with a threshold of d > 2 yielded 10 and 21 proteins in the whole serum and sEV samples, respectively. In the ROC analyses (Step 6) 10 whole serum proteins (MMP9, HSPB1, CASP14, HBG1, IGHG4, DEFA1, VWF, HNRNPA1, S100A8, TLN1) and 17 sEV proteins (MMP9, HSPB1, CASP14, HBG1, FGB, GGCT, PF4, S100A7, FN1, ANPEP, FLG2, HSPA8, IGLL1, MMRN1, S100A14, SBSN, SPRR2E) were found to meet the AUC = 1 selection criteria. Table S3 includes the UniProt ID, Gene symbol, ratio of intensity means > 2 or < 0.5 and Cohen’s d effect size > 2 parameters for the selected proteins. The two sample groups shared four significantly altered proteins (highlighted in Table S3), namely MMP9, CASP14, HBG1 and HSPB1.

Following protein selection, PCA (Step 7) was performed to visualize the dataset, where several potentially correlated proteins were projected into a smaller number of variables. K-means clustering (Step 8) on the whole serum PCA biplot resulted in 3 inhomogeneous or incomplete clusters. Calculated cluster homogeneity and completeness scores are 0.56 and 0.73, respectively. In contrast to whole serum samples, the clustering of sEV samples formed homogeneous and complete clusters, with homogeneity and completeness scores of 1. The results of the PCA analyses and k-means clustering indicate considerable differences between the whole serum and sEV samples (Figure 2B). We found that the accuracy of distinguishing between various CNS tumors can be increased using a protein panel from serum-derived sEVs, compared to analyzing whole serum samples.

### 2.3. Statistical Evaluation and IPA of LC-MS Data Revealed the Background of Suitability Differences between Whole Serum and sEV Samples

#### 2.3.1. Quantitative Changes of the Proteome May Affect the Suitability of sEV Samples to Provide Biomarkers for CNS Tumor Status Monitoring

**Figure 3:**
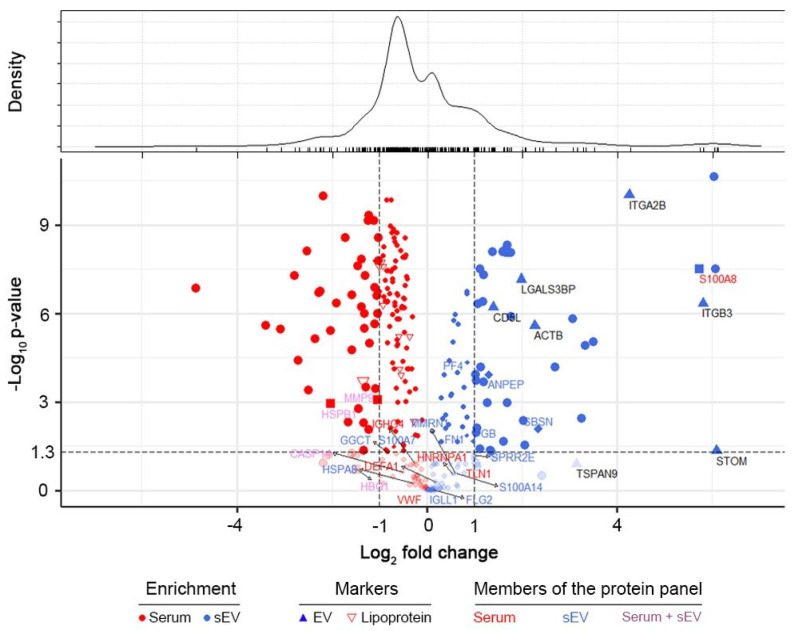
Quantitative comparison of the proteome of sEV and whole serum samples. Volcano plot represents the observed changes in average MS intensities in paired sEV vs. serum comparisons. Protein enrichment is marked with red and blue colored symbols in whole serum and sEVs, respectively. Lipoproteins (empty red upside-down triangles), elements of our whole serum protein panel (red letters, square symbols), sEV protein panel (blue letters, diamond symbols) and common members of the two protein panels (purple letters) are highlighted. Values of –log (p) were obtained from paired Welch’s test in sEV/serum comparisons. Density estimation of log2 (fold change) values is shown on top.

Statistical comparison of the proteome of sEV and whole serum samples was performed to reveal quantitative differences affecting the suitability of different sample types to provide biomarkers for CNS tumor status monitoring. Pairwise statistical comparison (Welch’s test) was used to identify proteins significantly enriched or depleted in sEV samples compared to whole serum samples (Figure 3). Sixty-five proteins were found to be significantly enriched in sEV samples, while 129 proteins were significantly depleted (*p* < 0.05). Using our sEV purification protocol detailed in the Section 4, we obtained a uniform particle size range of sEVs but the magnitude of quantitative changes in the sEV versus whole serum proteome suggested the possible presence of lipoprotein and serum protein contaminations. The level of apolipoproteins was decreased in sEV enriched samples (sEV/serum mean ratio is 0.66), however this fraction could not be completely eliminated. Besides, well known high abundance serum proteins (e.g., ALB) dominated the protein content of sEV enriched samples too. However, the enrichment of non-tissue specific (ITGA2B, ITGB3, LGALS3BP), epithelial cell (CD5L) and platelet related (STOM, TSPAN9) EV marker proteins [31] confirms sEV enrichment (sEV/serum mean ratio is 26.58), while it also demonstrates the presence of sEVs produced during clotting.

Among the 17 proteins of the sEV marker panel described in Section 2.2 only 6 were significantly enriched in the sEV samples and 5 of the 10 proteins comprising the specific serum panel had higher abundance in whole serum (Figure 3). These findings suggest that the better suitability of sEV enriched samples to serve a biomarker source is not explained by a total increase in the abundance of specific proteins. (Detailed proteomics findings, protein annotation and sEV enrichment data are available in Table S1).

Additional sample processing (sEV isolation) may introduce higher technical variance in case of sEV samples, thus it may reduce the analytical suitability of this sample type. Our analysis revealed a similar level of variance for proteins quantified in each sample type (excluding contaminants)—median coefficients of variation within each patient group were in the ranges of 20.78–23.87% for sEV and 20.21–24.45% for serum samples (see Figure S2 for CV distributions).

#### 2.3.2. Biological Background Might Be Responsible for the Increased Suitability of sEV Samples to Provide Biomarkers for CNS Tumor Status Monitoring

**Figure 4:**
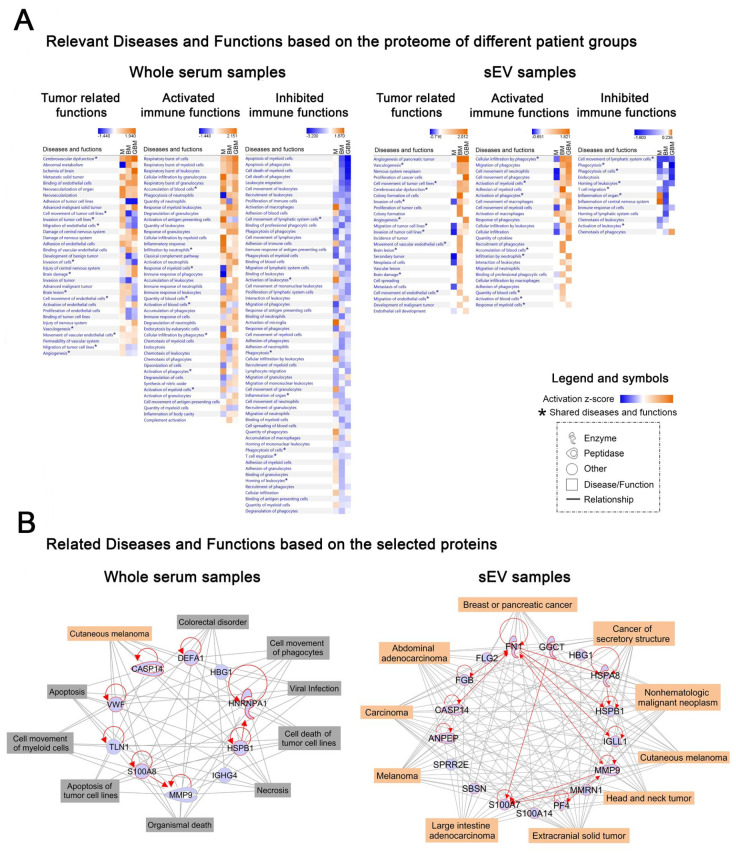
Ingenuity Pathway Analysis (IPA) analyses of whole serum (left) and sEV (right) data derived from the LC-MS analysis. (**A**) Heatmaps show relevant ‘Diseases and Functions’ in three separated panels related to systemic and tumor-related functions, as well as activated and inhibited immune functions. Z-score indicates activation or inhibition rates of the relevant ‘Diseases and Functions‘ in the three tumorous patient groups compared to the control group. * symbol indicates the shared diseases and functions in whole serum and sEVs. (**B**) Networks display the selected 10 whole serum or 17 sEV proteins (blue symbols) and their relationships (red lines). Top ten related ‘Diseases (highlighted in orange symbols) and Functions (highlighted in grey)’ are connected by grey lines.

To gain insight into the biological background of the obtained proteomics data, IPAwas applied. We performed ‘Core Analyses’ for whole serum and sEV data separately, yielding a list of significantly influenced ‘Diseases and Functions’ in each patient group (*p* < 0.05). Using ‘Comparison Analysis,’ we were able to develop heatmaps covering the relevant systemic and tumor-related functions, as well as the activated or inhibited immune functions (Figure 4A). Regarding whole serum samples, many of the significantly influenced functions identified are related to CNS involvement and active immune regulatory processes but the patient groups are not clearly distinguished on the heatmaps (Figure 4A, left panels). In contrast, on two of the three sEV proteome-based heatmaps M was evidently separated from the malignant tumor groups (Figure 4A, right panels), where tumor progression-related functions (e.g., angiogenesis, proliferation and migration of tumor cells) were detected to be highly activated and the activated immune functions (e.g., cell movement or activation of myeloid cells) predominate over inhibited immune functions (e.g., phagocytosis).

Next, we attempted to specify the common biological role of the characteristic protein profiles identified. Therefore, we elaborated two networks containing the selected 10 and 17 proteins identified based on whole serum and the sEV data, respectively (Figure 4B). Using the ‘Grow tool,’ the top ten influenced ‘Diseases and Functions’ were integrated into the networks. In case of the whole serum network (Figure 4B, left panel), nine different related ‘Diseases and Functions’ were identified, including viral infection, apoptosis, necrosis or cell movement of phagocytes and myeloid cells and only one was cancer-related. In contrast, the top ten influenced diseases identified on the sEV network based on the identified 17 proteins (Figure 4B, right panel) were all tumor-associated, suggesting their potential involvement in the pathophysiology of cancers.

## 3. Discussion

Non-invasive diagnostic tests are of outstanding clinical importance because of their minimal burden and risk to the patient, their repeatability, low cost, high information content and easy accessibility. In CNS tumors, a minimally invasive technique for describing the actual tumor status should be particularly important. Conventional MRI tests commonly used for the monitoring of CNS tumors are not absolutely appropriate for discriminating between various tumor types (e.g., cannot differentiate between glioblastomas and solitary metastases, CNS lymphomas or other glioma grades [32]) and cannot distinguish recurrence from pseudoprogression. Brain biopsies, as another option, are highly challenging and risky, especially when multiple sampling is required for long-term follow-up [33]. For several cancer types, blood-based tumor markers, such as PSA, AFP and CA125 have been introduced into clinical practice and research for the identification of further noninvasive biomarkers applicable for monitoring a wider scale of malignant diseases is ongoing [34]. However, regarding CNS tumors these studies have generally failed, presumably explained by several reasons, including (1) the barrier function of BBB (releasing less tumor ‘information’ into the systemic circulation), (2) the presence of molecules released into the blood from other sources and (3) possibly because of the complexity of tumor tissues (such as glioblastoma multiforme). These issues hamper attempts to use a single or only a few biomarkers to diagnose and monitor CNS tumors.

Based on these considerations, we aimed to detect the characteristic protein fingerprint of some common CNS tumors, trying to amplify the signal/information that brain tumors release into the circulation. For this purpose, the protein content of 96 clinical serum samples and related sEV samples isolated from the whole serum was measured by LC-MS. Serum samples were collected from three brain tumor groups considered as the most common malignant, benign and metastatic brain tumors (glioblastoma multiforme, meningioma [28] and brain metastasis of non-small-cell lung cancer [29]) and a control group (lumbar disc herniation).

To examine whether the proteomes of serum and sEV samples are suitable for differentiating between the CNS tumors in point, that is, whether they are applicable to diagnose and monitor the disease, the proteomes of these four patient groups were compared. The effectiveness of tumor type distinction may be increased if the analysis is restricted to proteins which exhibit significant between-group differences. Protein selection was carried out as described in literature [35] (using ratio of intensity means; Cohen’s d effect size; ROC) but much stricter thresholds were applied (>2, <0.5; d > 2; AUC = 1, respectively). Statistical selection yielded a collection of proteins whose intensity showed significant between-group differences and thus these proteins could be reckoned as the most suitable molecules for distinguishing between the tumor types examined. Specifically, protein selection yielded a 10- and 17-membered protein panel for whole serum and sEV samples, respectively. While none of these proteins appeared to be able to distinguish between the patient groups individually, their combination was found to reliably discriminate between the different patient groups suggesting that instead of a few candidates, a specific protein panel is required for a perfect differentiation between various tumor types.

To evaluate group distinction efficiency, PCA with k-means clustering was carried out according to literature [36]. Homogeneity and completeness scores of the clusters were calculated to measure the performance of k-means clustering. Cluster homogeneity and completeness mean that each cluster contains only samples from the same group and all samples of a given group are assigned to the same cluster. Both scores are bounded below by 0 and above by 1. A score of 1 indicates perfect homogeneity or completeness. PCA revealed that sEV samples were more suitable for group distinction. Despite carefully selected and perfectly identical statistical analyses for the two sample types, the homogeneity and completeness scores for the whole serum analysis were 0.56 and 0.73, respectively, compared to scores 1 and 1 for the analysis of sEV samples. The explanation for these findings is illustrated on a PCA biplot (Figure 2). Regarding serum samples, the proteins that can separate two given groups by the appropriate ratio and effect size may have similar intensities in other groups as well. For example, DEFA1 is important in distinguishing the CTRL group from the BM and GBM groups, however, it shows similar intensities in the GBM and BM groups, hampering the separation of these groups (see DEFA1 arrow pointing between the BM and GBM groups in the whole serum PCA plot). Still, DEFA1 cannot be removed, because it plays a key role in separating the CTRL group from malignant tumors. In contrast, the majority of the proteins identified in the sEV samples were able to separate any given group from all the others.

To check whether the poorer performance of whole serum proteins in distinguishing between the patient groups is attributed to the number of the proteins considered, we performed another PCA analysis including only up- and down-regulated proteins selected from the whole identified panel (see Figure 2, Step 4), yielding a similar number of proteins for the two sample types. The PCA analysis of these 41 whole serum and 45 sEV proteins yielded similar results as the previous analysis of carefully selected proteins only and the sEV sample type proved to perform better again. Although the sEV sample was far from being perfect in this case (4 groups were recognized with a homogeneity score of 0.66 and a completeness score of 0.66), the results for the whole serum analysis indicated that not even the sample groups can be recognized based on these proteins only 2 groups were recognized, homogeneity—0.07, completeness: 0.40) (Figure S3). These findings support that sEVs have a better efficiency in distinguishing between various patient groups, irrespective of the order of magnitude of proteins analyzed for the comparison of sEV and whole serum samples.

To investigate the background of our observations, we performed a quantitative proteomics comparison of the two sample types. A quantitative evaluation of sEV purification protocols was suggested based on quantitative LC-MS based proteomics approach, using enrichment analysis of carefully selected sEV markers along with medium specific contamination marker proteins (e.g., lipoproteins and serum). To the best of our knowledge, we are the first group to quantitatively compare the proteome of serum derived small extracellular vesicles with that of the original whole serum samples. sEV enrichment may increase the relative abundance of proteins present in higher concentration within sEVs and the increased signal-to-noise ratio may be beneficial for the quantitative LC-MS analysis of such proteins. On the other hand, proteins in serum are originating from different sources of the human body. Any fractionation (e.g., enrichment of a specific sEV population) may decrease the suppressing effect of the uninformative protein fraction released from sources not specific for the target disease. No association was revealed between being a sEV marker and sEV enrichment, suggesting that it not the overall enrichment process that should be responsible for the increased suitability of sEV samples to provide biomarkers for CNS tumor monitoring. Instead, the removal of an uninformative protein fraction, providing a more specific sample, may explain why the sEV sample is more applicable for distinguishing between various CNS cancer patient groups. Compared to whole serum samples, EVs may be more suitable for investigating tumor related molecular patterns, as the characteristic fingerprint molecules are present in higher concentrations in sEV samples and are accompanied by less contaminating molecules that may bias the analytical findings.

To understand the biological background for our proteomics-based data, IPA was used for the separate analyses of whole serum and sEV data. ‘Core Analyses’ were performed, yielding a list of significantly influenced ‘Diseases and Functions’ comprising tumor-related functions as well as activated or inhibited immune functions in each patient group (*p* < 0.05). ‘Comparison Analysis‘ was carried out to compare the affected ‘Diseases and Functions’ in the different patient groups. Regarding whole serum samples, many of the significantly influenced functions identified were associated with CNS involvement and active immune regulatory processes but the patient groups were not clearly distinguished on the heatmaps. In contrast, on the sEV proteome-based heatmaps the benign M was clearly separated from the malignant tumors, for which numerous tumor progression-related functions (e.g., angiogenesis, proliferation and migration of tumor cells) were found to be highly activated. The generated IPA heatmaps also revealed that the proteome of sEV samples may provide more specific information on the immune reactions characteristic to the patient groups. We assume that activated immune functions (e.g., cellular infiltration and migration of phagocytes) may play a crucial role in the development of an immune-suppressive microenvironment, while antitumoral immune responses (e.g., phagocytosis, inflammation) might be inhibited.

Serum is a dual source of biomolecular information on cancer, as it contains the molecules released by cancer cells, as well as those released during the immune system’s tumor-specific responses [37]. Therefore, the differences observed in the serum vesicles isolated from different patient groups may not only mirror tumor-specific processes but also those related to the associated immune responses [38,39]. Samples enriched in sEVs can offer an amplified source of relevant information, representing not only the specific tumor tissue but also the associated immune responses. Thus, an appropriate protein panel, covering both sources, may have improved efficiency for CNS tumor classification and monitoring.

In addition, the networks developed based on the IPA ‘Grow tool‘ demonstrated that the biological background of the sEV-based characteristic protein profile is more specifically associated with the tumor types compared with the whole serum based protein profile. The role of some of the proteins included in the sEV-based characteristic protein profile has already been described, for example in GBM biology, making these proteins promising targets for extracellular vesicle-based biomarker development [25].

In addition to the proteomics-based comparison of EV samples, we also examined the EV concentration of individual serum samples. Interestingly, no significant differences were detected between the four patient groups regarding the concentration of serum sEVs, with a mean size of 112.2 nm. Osti et al. observed higher EV plasma levels in GBM patients, brain metastases and extra-axial brain tumors compared to healthy controls. Other researchers also demonstrated higher EV concentration in tumorous patients, when unfractionated EV isolates [40] or a wider spectra of EVs were analyzed [41,42]. However, other non-neoplastic diseases of the central nervous system may also increase the number of small-sized circulating EVs, as it was demonstrated in acute ischemic stroke [43] or multiple sclerosis patients [44]. Our vesicle number measurement results, as well as the findings detailed above suggest that the elevated sEV concentration cannot be clearly attributed to the presence of the tumor as immune responses or other systemic responses also contribute to the circulating EV population. Our proteomics-based findings, coupled with the available literature data, suggest that circulating small-sized EVs show important qualitative but not quantitative differences between benign or malignant brain tumors and spinal disc herniation.

Liu and colleagues highlighted that serum is not the perfect choice for a representative sampling of circulating EVs [45], as a high fraction of EVs may be lost during coagulation and also blood components (e.g., platelets) may release microvesicles (MV) during clotting, altering the original MV content of blood samples. However, serum is still the preferred sample form for blood-based clinical diagnoses and it is a practical choice for future clinical developments. It should be noted that co-purification of proteins [46] and lipoprotein particles [47] in EV isolation methods is a common and well known challenge [31]. The presence of protein aggregates [48] and lipoproteins in sEV isolates may provide additional explanation for the lack of increase in the concentration of enriched sEV particles in cancer patients’ serum, contrary to literature data on plasma [49] or serum samples [40]. Efforts to eliminate lipoproteins are described in numerous papers reporting on attempts to introduce more sophisticated methods (e.g., combination of ultracentrifugation and size-exclusion chromatography) [50]. In fact, these laborious and instrumentation demanding methods are of high importance in scientific research of the molecular contents of EVs but they may not be applicable in routine clinical practice. Our sEV isolation protocol has several advantages, as it does not require expensive equipment or highly trained professionals and the entire procedure (along with characterization) is performed within 4 h, therefore, this technique could be easily incorporated into clinical practice.

Our quantitative proteomics results demonstrate that even a simple sEV enrichment protocol can increase the diagnostic potential of serum samples for the identification and classification of patients with different CNS cancers. This finding also supports that even a low-efficacy sEV enrichment/purification method may be appropriate to enhance the analytical applicability of serum samples for CNS cancer monitoring, however, in such cases a quantitative description of enrichment efficiency is definitely required for the right interpretation of the analytical results [51].

In conclusion, our findings support that extracellular vesicles have a greater potential for the monitoring of CNS tumors compared to whole serum samples. Using EV samples is a possible way to amplify the signals released by brain tumors into the circulation. Given the easy-to-implement isolation and enrichment protocol established in this study, the introduction of EV analysis would be beneficial in clinical practice.

## 4. Materials and Methods

### 4.1. Patients

Blood samples of 96 patients treated between March 2015 and January 2018 in the Department of Neurosurgery, University of Debrecen were analyzed. Samples were obtained from patients with primary glioblastoma multiforme (GBM), meningioma (M) and single brain metastasis originating from non-small-cell lung cancer (BM). Control samples (CTRL) were collected from patients with spinal disc herniation without evidence of cancer. This non-tumor patient group served as control group in comparison to the patients having different intracranial tumor to distinguish the effects of tumorous processes from the CNS involvement on circulating sEVs. Each group contained 24 individuals with mixed ages and genders. As shown in the Table 1, six-sample-pools were created from the individuals, allowing four parallel samples to be tested per group. Blood samples were collected one day prior to neurosurgical procedure in each tumor case. None of the patients received radio- or chemotherapy before tumor resection. Blood samples were stored by the Neurosurgical Brain Tumor and Tissue Bank of Debrecen according to the criteria of the National Research Ethics Committee. An informed consent form was signed by each patient; the study was conducted in accordance with the Declaration of Helsinki. This study was carried out according to two ethical approvals, namely 51450-2/2015/EKU (0411/15), Medical Research Council, Scientific and Research Ethics Committee, Budapest, October 30, 2015 and 121/2019-SZTE, University of Szeged, Human Investigation Review Board, Albert Szent-Györgyi Clinical Centre, Szeged, 19 July 2019.

### 4.2. Preparation of Serum Samples, sEV Isolation and Characterization

Blood samples were collected into BD Vacutainer SST II Advance Tubes (Becton, Dickinson and Company, Franklin Lakes, NJ, USA), allowed to clot for at least 1 h at room temperature and centrifuged for 20 min at 3000× *g*, 10 °C to remove cells. Following the 3000× *g* centrifugation, the supernatant serum was transferred to new Eppendorf tubes and centrifuged for 30 min at 10,000× *g*, 4 °C to remove debris and large vesicles. One milliliter serum aliquot was diluted with DPBS (Ca^2+^-free, Mg^2+^-free, Lonza Group Ltd., Basel, Switzerland) to 8 mL and ultracentrifuged for 70 min, at 100,000× *g*, 4 °C (polycarbonate tubes, fixed angle T-1270 rotor, Thermo Fisher Scientific, Waltham, MA, USA). The pellet was resuspended in 100 uL DPBS and stored at −80 °C until further processing. This sEV isolation protocol served to reach intermediate recovery and intermediate specificity according to MISEV2018 [31].

SEVs were characterized by AFM (Oxford Instruments Asylum Research, Abingdon, UK), as described previously [52] and NTA using a NanoSight NS300 instrument (Malvern Panalytical Ltd., Malvern, UK) as it described below. Classical EV markers were presented by Western blot analyses using NuPAGE reagents and an XCell SureLock Mini-Cell System (Thermo Fisher Scientific, Waltham, MA, USA) according to manufacturer’s protocols. For detection of the CD81 and Alix markers, we used rabbit anti-human CD81 (1:1000, Sigma-Aldrich, St. Louis, MO, USA) and rabbit anti-human Alix (1:1000, Sigma-Aldrich, St. Louis, MO, USA) primary antibody and HRP-conjugated anti-rabbit IgG (1:1000, R&D Systems, Minneapolis, MN, USA) secondary antibody.4.3. Quantitative analysis of sEVs by NTA.

As suggested by a recent study [53], sEVs were diluted in particle free DPBS and analyzed using a NanoSight NS300 instrument with 532 nm laser (Malvern Panalytical Ltd., Malvern, UK). The measurements were performed on the 16 sEV sample pools (described in 4.1). Six videos of 60 s were recorded for each sample under constant settings (Camera level: 15; Treshold: 4, 25 °C; 60–80 particles/frame) and analyzed to obtain data on size distribution and particle concentration.

### 4.3. Proteomic Analysis by LC-MS

#### 4.3.1. ‘In Solution’ Digestion

Individual samples containing 20 µg protein were diluted to 10 µL with 0.1 M NH_4_HCO_3_ (pH = 8.0) buffer; 12 µL 0.1% RapiGest SF (Waters, Milford, MA, USA) and 2 µL 55 mM dithioeritritol solution was added and kept at 60 °C for 30 min to unfold and reduce proteins. A volume of 2 µL 200 mM iodo acetamide solution was added to alkylate the proteins which were kept for an additional 30 min in the dark at room temperature. The samples were digested overnight at 37 °C with trypsin (Thermo Scientific, Waltham, MA, USA, enzyme/protein ratio: 0.4 to 1). The digestion was stopped by addition of 1 μL of concentrated formic acid.

#### 4.3.2. LC-MS

The separation of the digested samples was carried out on a nanoAcquity UPLC, (Waters, Milford, MA, USA) using Waters ACQUITY UPLC M-Class Peptide C18 (130 Å, 1.78 μm, 75 μm × 250 mm) column with a nonlinear 90 min gradient. Eluents were water (A) and acetonitrile (B) containing 0.1 *V*/*V*% formic acid and the separation of the peptide mixture was performed at 45 °C with 0.35 μL/min flow rate using an optimized nonlinear LC gradient (3–40% B). The LC was coupled to a high-resolution Q Exactive Plus quadrupole-orbitrap hybrid mass spectrometer (Thermo Scientific, Waltham, MA, USA). The quantitative measurements of digested individual samples were performed in DIA mode. The survey scan for DIA method operated with 35,000 resolution. The full scan was performed between 380 to 1020 *m*/*z*. The AGC target was set to 5 × 106 or 120 ms maximum injection time. In the 400–1000 *m*/*z* region 22 *m*/*z* wide overlapping windows were acquired at 17,500 resolution (AGC target: 3 × 106 or 100 ms injection time, normalized collision energy: 27 for charge 2). The quantitative analysis was performed in Encyclopedia 0.81 [54] using default settings after deconvolution, peak picking and conversion of raw MS files to mzML format in Proteowizard [55]. A comprehensive spectral library [56] of 10,000 human proteins was used for peptide identification. Protein quantities calculated by the Encyclopedia software based on summed intensities of the automatically filtered peptides were used in further statistical evaluations.

### 4.4. Statistical Analysis

The collected data about the whole serum and extracellular vesicles were reduced and analyzed using statistical methods. Pearson’s correlation analysis was used to investigate the outlier samples [57], contaminating proteins (cytokeratins) and proteins with missing values were excluded from the proteomic data [58]. Data were log-transformed to reduce skewness and increase linearity [59]. Cohen’s d effect size was calculated to measure the difference between the protein intensity means, outcomes in two different groups [60,61]. Pairwise ROC analysis allowed us to find those proteins which can separate at least one group to the others [62]. The calculated ROC AUC (area under the ROC curve) values are accepted if it equals to 1. In order to transform several (potentially) correlated proteins into a (smaller) number of uncorrelated variables and visualize the dataset, PCA with k-means clustering was performed [63,64,65,66]. Homogeneity and completeness scores of the clusters were calculated to measure the performance of k-means clustering [67]. Two-tailed Welch’s *t*-test was performed to identify the significantly enriched or depleted proteins in sEV samples. The statistical analyses were performed using R statistical program (version 3.6.3 with pROC, FactoMineR, factoextra and ggplot2 packages; Vienna, Austria), Python programming language (version 3.8, Scotts Valley, CA, USA) and Perseus (MaxQuant, Munich, Germany). Values of *p* < 0.05 were considered significant (see in Appendix A more detailed). GraphPad Prism 8 (San Diego, CA, USA) was used for further data visualization.

### 4.5. In Silico Analysis of LC-MS Data

Protein data derived from the LC-MS were analyzed by the IPA (Qiagen Bioinformatics, Hilden, Germany). Using fold change values, ‘Core Analysis’ were performed for whole serum and sEV data separately to identify ‘Diseases and Functions,’ which can be significantly influenced by the described proteomes (*p* < 0.05). After ‘Comparison Analysis,’ we created heatmaps of the relevant ‘Diseases and Functions,’ that is, tumor-related and immunological functions showing regulatory differences between the three CNS tumor groups. Activation z-score calculated by IPA indicates the extent and direction of the effect that given proteins have on function/disease.

The selected 10 whole serum proteins and 17 sEV proteins were introduced to custom pathways as well. Then, ‘Connect tool’ of IPA was used to reveal the relationships between these molecules and ‘Grow tool’ was applied to search the top ten ‘Diseases and Functions’ assigned to the 10 whole serum proteins or 17 sEV proteins. Results are displayed in two networks created by the IPA Path Designer.

Confidence level was set to ‘Experimentally observed’ for all IPA procedures, which enables literature data-based analysis but excludes unproven predictions.

### 4.6. Data Availability

All datasets generated during the current study are available from the corresponding author upon reasonable request.

We have submitted all relevant data of our experiments to the EV-TRACK [68] knowledgebase EV-TRACK ID: EV200080.

## 5. Conclusions

Our study aimed to detect the characteristic protein fingerprint of the most common CNS tumors. Intending to amplify the signal that brain tumors release into the circulation, in addition to the whole serum’s, the protein content of the small extracellular vesicles isolated from the serum was also examined.

Comparative proteomic analysis suggests that sEVs may be more suitable for investigating tumor related molecular patterns, because these molecules are present in higher concentrations in sEV samples compared to whole serum samples and have less ‘noise’ that may bias the analytical findings. In silico analyses revealed that the biological background of the sEV-based characteristic protein profile of the samples is more specifically associated with the tumor types compared with the whole serum based protein profile. Samples enriched in sEVs can offer an amplified source of relevant information, representing not only the specific tumor tissue but also the associated immune responses.

These findings revealed that circulating small-sized extracellular vesicles were more suitable for separating different patient groups. The number of proteins applied for monitoring cannot be reduced to a few individual molecules, instead, a specific protein panel is required for perfect differentiation. To the best of our knowledge, we are the first group to quantitatively compare the proteome of serum derived small extracellular vesicles with that of the original whole serum samples.

In conclusion, our findings support that extracellular vesicles have a greater potential for monitoring CNS tumors, compared to whole serum samples. Considering that analyzing sEVs can be performed easily, incorporating our method into clinical practice would be of great benefit.

## Figures and Tables

**Table 1:** Patient cohort ^1^.

**♦ Glioblastoma Multiforme**	**GBM**	**GBM1**	**GBM2**	**GBM3**	**GBM4**
Total No. of patients	***n* = 24**	*n* = 6	*n* = 6	*n* = 6	*n* = 6
Age, Median (range) Mean	**67 (33–82)** **64.9**	64.5 (38–82) 62.7	69.5 (33–76) 63.8	67.5 (49–74) 64.7	66.5 (63–77) 68.5
Sex (%), Male Female	**13 (54.2)** **11 (45.8)**	3 (50) 3 (50)	3 (50) 3 (50)	5 (83.3) 1 (16.7)	2 (33.3) 4 (66.7)
**■ Brain Metastasis**	**BM**	**BM1**	**BM2**	**BM3**	**BM4**
Total No. of patients	***n* = 24**	*n* = 6	*n* = 6	*n* = 6	*n* = 6
Age, Median (range) Mean	**64 (42–82)** **64**	66.5 (51–82) 67.7	68 (62–71) 67.5	63.5 (42–81) 59.7	59.5 (53–64) 59.5
Sex (%), Male Female	**13 (54.2)** **11 (45.8)**	2 (33.3) 4 (66.7)	3 (50) 3 (50)	4 (66.7) 2 (33.3)	4 (66.7) 2 (33.3)
**▲ Meningioma**	**M**	**M1**	**M2**	**M3**	**M4**
Total No. of patients	***n* = 24**	*n* = 6	*n* = 6	*n* = 6	*n* = 6
Age, Median (range) Mean	**60 (30–79)** **58.0**	54.5 (39–69) 53.5	62 (30–66) 53	61.5 (44–75) 59.3	66.5 (52–79) 66
Sex (%), Male Female	**4 (16.7)** **20 (83.3)**	0 (0) 6 (100)	0 (0) 6 (100)	1 (16.7) 5 (83.3)	3 (50) 3 (50)
**● Control**	**CTRL**	**CTRL1**	**CTRL2**	**CTRL3**	**CTRL4**
Total No. of patients	***n* = 24**	*n* = 6	*n* = 6	*n* = 6	*n* = 6
Age, Median (range) Mean	**50.5 (20–81)** **52.9**	46.5 (26–71) 46.5	47 (20–62) 45	70.5 (49–81) 67.2	52.5 (41–69) 53
Sex (%), Male Female	**9 (37.5)** **15 (62.5)**	2 (33.3) 4 (66.7)	4 (66.7) 2 (33.3)	4 (66.7) 2 (33.3)	4 (66.7) 2 (33.3)

^1^ The table summarizes the main characteristics of the patient groups examined. Each group (average values highlighted in bald) included 24 individuals, converted into six-sample-pools to yield four samples per group for further analysis.

## Supplementary Materials

Supplementary materials can be found at https://www.mdpi.com/1422-0067/21/15/5359/s1. Figure S1: Western blot analyses of classical EV markers, Figure S2: Intragroup Coefficients of variation (CV) distributions, Figure S3: PCA dotplot constructed after statistical selection based on the means of intensity ratio; Table S1: 311 membered protein table of DIA mode constructed spectral library; Table S2: Sample correlation matrix; Table S3: List of the selected proteins.

## Abbreviations

AFM	Atomic force microscopy
BBB	Blood brain barrier
BM	Brain metastasis originating from non-small-cell lung cancer
CNS	Central nervous system
CTRL	Controls – lumbar disc hernia patients
DIA	Data independent acquisition
EVs	Extracellular Vesicles
GBM	Primary glioblastoma multiforme
IPA	Ingenuity Pathway Analysis
LC-MS	Liquid chromatography - mass spectrometry
M	Meningioma
NTA	Nanoparticle tracking analysis
PCA	Principal component analysis
ROC	Receiver operating characteristic
sEVs	small extracellular vesicles

## Appendix A. Detailed Description of the Statistical Analyses.

The collected data about the whole serum and extracellular vesicles were reduced and analyzed using statistical methods. Pearson’s correlation analysis was used to investigate the outlier samples [57]. Contaminating proteins (cytokeratins) and proteins with missing values were excluded from the proteomic data [58]. Data were log-transformed to reduce skewness and increase linearity [59].

Cohen’s d effect size was calculated to measure the difference between the protein intensity mean, outcomes in two different groups. The formula of the Cohen’s d effect size

$$d=\frac{\left|{\bar{X}}_{1}-{\bar{X}}_{2}\right|}{\sqrt{\frac{\left(n_{1}-1\right)SD_{1}^{2}+\left(n_{2}-1\right)SD_{2}^{2}}{n_{1}+n_{2}-2}}}$$

where *X* is the mean protein intensity in a given group, *SD* is standard deviation and n is sample size [60]. In this study we say at least 2 effect size is necessary. It indicates that the mean of group 1 is at the 97.7 percentile of group 2, and the nonoverlapping area of the two distributions at least is 81.1% [61].

Pairwise ROC analysis allowed us to find those proteins which can separate at least one group to the others [62]. The ROC analysis use the true positive rate (sensitivity) and the true negative rate (specificity) at various threshold settings. Plotting the sensitivity against the 1-specificity we get the ROC curve, under the area under this curve measure the separability of the given variable (protein). AUC = 0.5 represents an unsuitable variable to the separate two groups. If AUC = 1, the separation using the actual variable is error-free. In our study the calculated AUC (area under the curve) values are accepted if it equals to 1.

In order to transform several (potentially) correlated proteins into a (smaller) number of uncorrelated variables, and visualize the dataset, principal component analysis (PCA) with k-means clustering was performed. The goal of PCA is to reduce a large number of correlated variables with a set of uncorrelated principal components. These components can be thought of as linear combinations of the original variables that are optimally weighted and derived from the correlation matrix of the data [63].

K-means clustering was performed on the obtained PCA score plots by Hartigan-Wong algorithm [64,65]. Optimal numbers of clusters were determined with Silhouette method and these recommended values were used for clustering [66].

Homogeneity and completeness scores of the clusters were calculated to measure the performance of k-means clustering Cluster homogeneity and completeness mean that each cluster contains only samples from the same group, and all samples of a given group are assigned to the same cluster. Both scores are bounded below by 0 and above by 1. A score of 1 indicates the perfect homogeneity or completeness. [67].

Two-tailed Welch’s t-test was performed to identify significantly enriched or depleted proteins in sEV samples.

The statistical analyses were performed using R statistical program (version 3.6.3 with pROC, FactoMineR, factoextra and ggplot2 packages), Python programming language (version 3.8) and Perseus (MaxQuant). Values of *p* < 0.05 were considered significant. GraphPad Prism 8 was used for further data visualization.

## References

[1] World Health Organization (2018). WHO Guidelines for the Pharmacological and Radiotherapeutic Management of Cancer Pain in Adults and Adolescents.

[2] World Health Organization (2017). Guide to Cancer Early Diagnosis.

[3] Garden G.A., Campbell B.M. (2016). Glial biomarkers in human central nervous system disease. Glia.

[4] Staedtke V., Dzaye O., Holdhoff M. (2016). Actionable molecular biomarkers in primary brain tumors. Trends Cancer.

[5] Good D.M., Thongboonkerd V., Novak J., Bascands J.L., Schanstra J.P., Coon J.J., Dominiczak A., Mischak H. (2007). Body fluid proteomics for biomarker discovery: Lessons from the past hold the key to success in the future. J. Proteome Res..

[6] Best M.G., Sol N., Zijl S., Reijneveld J.C., Wesseling P., Wurdinger T. (2015). Liquid biopsies in patients with diffuse glioma. Acta Neuropathol..

[7] Gerber D.E., Grossman S.A., Zeltzman M., Parisi M.A., Kleinberg L. (2007). The impact of thrombocytopenia from temozolomide and radiation in newly diagnosed adults with high-grade gliomas. Neuro Oncol..

[8] Cagney D.N., Sul J., Huang R.Y., Ligon K.L., Wen P.Y., Alexander B.M. (2018). The FDA NIH Biomarkers, EndpointS and other Tools (BEST) resource in neuro-oncology. Neuro Oncol..

[9] Sheridan C. (2016). Exosome cancer diagnostic reaches market. Nat. Biotechnol..

[10] Colombo M., Raposo G., Théry C. (2014). Biogenesis, secretion and intercellular interactions of exosomes and other extracellular vesicles. Annu. Rev. Cell Dev. Biol.

[11] Nogués L., Benito-Martin A., Hergueta-Redondo M., Peinado H. (2018). The influence of tumour-derived extracellular vesicles on local and distal metastatic dissemination. Mol. Asp. Med..

[12] Hoshino A., Costa-Silva B., Shen T.L., Rodrigues G., Hashimoto A., Tesic Mark M., Molina H., Kohsaka S., Di Giannatale A., Ceder S. (2015). Tumour exosome integrins determine organotropic metastasis. Nature.

[13] Costa-Silva B., Aiello N.M., Ocean A.J., Singh S., Zhang H., Thakur B.K., Becker A., Hoshino A., Mark M.T., Molina H. (2015). Pancreatic cancer exosomes initiate pre-metastatic niche formation in the liver. Nat. Cell Biol..

[14] Liu Y., Gu Y., Han Y., Zhang Q., Jiang Z., Zhang X., Huang B., Xu X., Zheng J., Cao X. (2016). Tumor Exosomal RNAs Promote Lung Pre-metastatic Niche Formation by Activating Alveolar Epithelial TLR3 to Recruit Neutrophils. Cancer Cell.

[15] Zeng Z., Li Y., Pan Y., Lan X., Song F., Sun J., Zhou K., Liu X., Ren X., Wang F. (2018). Cancer-derived exosomal miR-25-3p promotes pre-metastatic niche formation by inducing vascular permeability and angiogenesis. Nat. Commun..

[16] Feng W., Dean D.C., Hornicek F.J., Shi H., Duan Z. (2019). Exosomes promote pre-metastatic niche formation in ovarian cancer. Mol. Cancer.

[17] Chen G., Huang A.C., Zhang W., Zhang G., Wu M., Xu W., Yu Z., Yang J., Wang B., Sun H. (2018). Exosomal PD-L1 contributes to immunosuppression and is associated with anti-PD-1 response. Nature.

[18] Scavo M.P., Depalo N., Tutino V., De Nunzio V., Ingrosso C., Rizzi F., Notarnicola M., Curri M.L., Giannelli G. (2020). Exosomes for Diagnosis and Therapy in Gastrointestinal Cancers. Int. J. Mol. Sci..

[19] Basu B., Ghosh M.K. (2019). Extracellular Vesicles in Glioma: From Diagnosis to Therapy. Bioessays.

[20] Kosaka N., Kogure A., Yamamoto T., Urabe F., Usuba W., Prieto-Vila M., Ochiya T. (2019). Exploiting the message from cancer: The diagnostic value of extracellular vesicles for clinical applications. Exp. Mol. Med..

[21] Möhrmann L., Huang H.J., Hong D.S., Tsimberidou A.M., Fu S., Piha-Paul S.A., Subbiah V., Karp D.D., Naing A., Krug A. (2018). Liquid biopsies using plasma exosomal nucleic acids and plasma cell-free DNA compared with clinical outcomes of patients with advanced cancers. Clin. Cancer Res..

[22] Melo S.A., Luecke L.B., Kahlert C., Fernandez A.F., Gammon S.T., Kaye J., LeBleu V.S., Mittendorf E.A., Weitz J., Rahbari N. (2015). Glypican-1 identifies cancer exosomes and detects early pancreatic cancer. Nature.

[23] Choy C., Jandial R. (2016). Breast Cancer Exosomes Breach the Blood-Brain Barrier. Neurosurgery.

[24] García-Romero N., Carrión-Navarro J., Esteban-Rubio S., Lázaro-Ibáñez E., Peris-Celda M., Alonso M.M., Guzmán-De-Villoria J., Fernández-Carballal C., de Mendivil A.O., García-Duque S. (2017). DNA sequences within glioma-derived extracellular vesicles can cross the intact blood-brain barrier and be detected in peripheral blood of patients. Oncotarget.

[25] Gollapalli K., Ray S., Srivastava R., Renu D., Singh P., Dhali S., Bajpai Dikshit J., Srikanth R., Moiyadi A., Srivastava S. (2012). Investigation of serum proteome alterations in human glioblastoma multiforme. Proteomics.

[26] Gállego Pérez-Larraya J., Paris S., Idbaih A., Dehais C., Laigle-Donadey F., Navarro S., Capelle L., Mokhtari K., Marie Y., Sanson M. (2014). Diagnostic and prognostic value of preoperative combined GFAP, IGFBP-2 and YKL-40 plasma levels in patients with glioblastoma. Cancer.

[27] Figueroa J.M., Carter B.S. (2018). Detection of glioblastoma in biofluids. J. Neurosurg..

[28] Ostrom Q.T., Gittleman H., Truitt G., Boscia A., Kruchko C., Barnholtz-Sloan J.S. (2018). CBTRUS Statistical Report: Primary Brain and Other Central Nervous System Tumors Diagnosed in the United States in 2011-2015. Neuro Oncol..

[29] Fox B.D., Cheung V.J., Patel A.J., Suki D., Rao G. (2011). Epidemiology of Metastatic Brain Tumors. Neurosurg. Clin. N. Am..

[30] Théry C., Witwer K.W., Aikawa E., Alcaraz M.J., Anderson J.D., Andriantsitohaina R., Antoniou A., Arab T., Archer F., Atkin-Smith G.K. (2018). Minimal information for studies of extracellular vesicles 2018 (MISEV2018): A position statement of the International Society for Extracellular Vesicles and update of the MISEV2014 guidelines. J. Extracell. Vesicles.

[31] de Menezes-Neto A., Sáez M.J., Lozano-Ramos I., Segui-Barber J., Martin-Jaular L., Ullate J.M., Fernandez-Becerra C., Borrás F.E., Del Portillo H.A. (2015). Size-exclusion chromatography as a stand-alone methodology identifies novel markers in mass spectrometry analyses of plasma-derived vesicles from healthy individuals. J. Extracell. Vesicles.

[32] Weber M.A., Zoubaa S., Schlieter M., Jüttler E., Huttner H.B., Geletneky K., Ittrich C., Lichy M.P., Kroll A., Debus J. (2006). Essig Diagnostic performance of spectroscopic and perfusion MRI for distinction of brain tumors. Neurology.

[33] Shankar G.M., Balaj L., Stott S.L., Nahed B., Carter B.S. (2017). Liquid biopsy for brain tumors. Expert Rev. Mol. Diagn..

[34] Marrugo-Ramírez J., Mir M., Samitier J. (2018). Blood-Based Cancer Biomarkers in Liquid Biopsy: A Promising Non-Invasive Alternative to Tissue Biopsy. Int. J. Mol. Sci..

[35] Miyauchi E., Furuta T., Ohtsuki S., Tachikawa M., Uchida Y., Sabit H., Obuchi W., Baba T., Watanabe M., Terasaki T. (2018). Identification of blood biomarkers in glioblastoma by SWATH mass spectrometry and quantitative targeted absolute proteomics. PLoS ONE.

[36] Rhie S.K., Perez A.A., Lay F.D., Schreiner S., Shi J., Polin J., Farnham P.J. (2019). A high-resolution 3D epigenomic map reveals insights into the creation of the prostate cancer transcriptome. Nat. Commun..

[37] Anderson K.S., LaBaer J. (2005). The sentinel within: Exploiting the immune system for cancer biomarkers. J. Proteome Res..

[38] Wen C., Seeger R.C., Fabbri M., Wang L., Wayne A.S., Jong A.Y. (2017). Biological roles and potential applications of immune cell-derived extracellular vesicles. J. Extracell. Vesicles.

[39] Veerman R.E., Güçlüler Akpinar G., Eldh M., Gabrielsson S. (2019). Immune Cell-Derived Extracellular Vesicles –Functions and Therapeutic Applications. Trends Mol. Med..

[40] Gercel-Taylor C., Atay S., Tullis R.H., Kesimer M., Taylor D.D. (2012). Nanoparticle analysis of circulating cell-derived vesicles in ovarian cancer patients. Anal. Biochem..

[41] Lázaro-Ibáñez E., Sanz-Garcia A., Visakorpi T., Escobedo-Lucea C., Siljander P., Ayuso-Sacido A., Yliperttula M. (2014). Different gDNA content in the subpopulations of prostate cancer extracellular vesicles: Apoptotic bodies, microvesicles and exosomes. Prostate.

[42] König L., Kasimir-Bauer S., Bittner A.K., Hoffmann O., Wagner B., Santos Manvailer L.F., Kimmig R., Horn P.A., Rebmann V. (2017). Elevated levels of extracellular vesicles are associated with therapy failure and disease progression in breast cancer patients undergoing neoadjuvant chemotherapy. Oncoimmunology.

[43] Ji Q., Ji Y., Peng J., Zhou X., Chen X., Zhao H., Xu T., Chen L., Xu Y. (2016). Increased Brain-Specific MiR-9 and MiR-124 in the Serum Exosomes of Acute Ischemic Stroke Patients. PLoS ONE.

[44] Galazka G., Mycko M.P., Selmaj I., Raine C.S., Selmaj K.W. (2018). Multiple sclerosis: Serum-derived exosomes express myelin proteins. Mult. Scler..

[45] Liu M.-L., Werth V.P., Williams K.J. (2020). Blood plasma versus serum: Which is right for sampling circulating membrane microvesicles in human subjects?. Ann. Rheum. Dis..

[46] Smolarz M., Pietrowska M., Matysiak N., Mielańczyk Ł., Widłak P. (2019). Proteome Profiling of Exosomes Purified from a Small Amount of Human Serum: The Problem of Co-Purified Serum Components. Proteomes.

[47] Sódar B.W., Kittel Á., Pálóczi K., Vukman K.V., Osteikoetxea X., Szabó-Taylor K., Németh A., Sperlágh B., Baranyai T., Giricz Z. (2016). Low-density lipoprotein mimics blood plasma-derived exosomes and microvesicles during isolation and detection. Sci. Rep..

[48] Filipe V., Hawe A., Jiskoot W. (2010). Critical evaluation of Nanoparticle Tracking Analysis (NTA) by NanoSight for the measurement of nanoparticles and protein aggregates. Pharm. Res..

[49] Osti D., Del Bene M., Rappa G., Santos M., Matafora V., Richichi C., Faletti S., Beznoussenko G.V., Mironov A., Bachi A. (2019). Clinical Significance of Extracellular Vesicles in Plasma from Glioblastoma Patients. Clin. Cancer Res..

[50] Karimi N., Cvjetkovic A., Jang S.C., Crescitelli R., Hosseinpour Feizi M.A., Nieuwland R., Lötvall J., Lässer C. (2018). Detailed analysis of the plasma extracellular vesicle proteome after separation from lipoproteins. Cell Mol. Life Sci..

[51] Xu R., Greening D.W., Zhu H.J., Takahashi N., Simpson R.J. (2016). Extracellular vesicle isolation and characterization: Toward clinical application. J. Clin. Investig..

[52] Harmati M., Tarnai Z., Decsi G., Kormondi S., Szegletes Z., Janovak L., Dekany I., Saydam O., Gyukity-Sebestyen E., Dobra G. (2017). Stressors alter intercellular communication and exosome profile of nasopharyngeal carcinoma cells. J. Oral Pathol. Med..

[53] Parsons M.E.M., McParland D., Szklanna P.B., Guang M.H.Z., O’Connell K., O’Connor H.D., McGuigan C., Ní Áinle F., McCann A., Maguire P.B. (2017). A Protocol for Improved Precision and Increased Confidence in Nanoparticle Tracking Analysis Concentration Measurements between 50 and 120 nm in Biological Fluids. Front. Cardiovasc. Med..

[54] Searle B.C., Pino L.K., Egertson J.D., Ting Y.S., Lawrence R.T., MacLean B.X., Villén J., MacCoss M.J. (2018). Chromatogram libraries improve peptide detection and quantification by data independent acquisition mass spectrometry. Nat. Commun..

[55] Chambers M., Maclean B., Burke R., Amodei D., Ruderman D.L., Neumann S., Gatto L., Fischer B., Pratt B., Egertson J. (2012). A cross-platform toolkit for mass spectrometry and proteomics. Nat. Biotechnol..

[56] Rosenberger G., Koh C.C., Guo T., Röst H.L., Kouvonen P., Collins B.C., Heusel M., Liu Y., Caron E., Vichalkovski A. (2014). A repository of assays to quantify 10,000 human proteins by SWATH-MS. Sci. Data.

[57] Metz T.O., Qian W.J., Jacobs J.M., Gritsenko M.A., Moore R.J., Polpitiya A.D., Monroe M.E., Camp D.G., Mueller P.W., Smith R.D. (2008). Application of proteomics in the discovery of candidate protein biomarkers in a diabetes autoantibody standardization program sample subset. J. Proteome Res..

[58] Hodge K., Have S.T., Hutton L., Lamond A.I. (2013). Cleaning up the masses: Exclusion lists to reduce contamination with HPLC-MS/MS. J. Proteom..

[59] Curran-Everett D. (2018). Explorations in statistics: The log transformation. Adv. Physiol. Educ..

[60] Lakens D. (2013). Calculating and reporting effect sizes to facilitate cumulative science: A practical primer for t-tests and ANOVAs. Front. Psychol..

[61] Cohen J. (1988). Statistical Power Analysis for the Behavioral Sciences.

[62] Fawcett T. (2006). An introduction to ROC analysis. Pattern Recognit. Lett..

[63] Husson F., Le S., Pagès J. (2017). Exploratory Multivariate Analysis by Example Using R.

[64] Hartigan J.A., Wong M.A. (1979). Algorithm AS 136: A K-Means Clustering Algorithm. J. Appl. Stat..

[65] Ding C., He X. (2004). K-means clustering via principal component analysis. Proceedings of the Twenty-First International Conference on Machine Learning.

[66] Rousseeuw P.J. (1987). Silhouettes: A graphical aid to the interpretation and validation of cluster analysis. J. Comput. Appl. Math..

[67] Pedregosa F., Varoquaux G., Gramfort A., Michel V., Thirion B., Grisel O., Blondel M., Prettenhofer P., Weiss R., Dubourg V. (2012). Scikit-learn: Machine Learning in Python. J. Mach. Learn. Res..

[68] Van Deun J., Mestdagh P., Agostinis P., Akay Ö., Anand S., Anckaert J., Martinez Z.A., Baetens T., Beghein E., Bertier L. (2017). EV-TRACK: Transparent reporting and centralizing knowledge in extracellular vesicle research. Nat. Methods.

