# Supplementary material for: Small Extracellular Vesicles Isolated from Serum May Serve as Signal-Enhancers for the Monitoring of CNS Tumors
Source: Int J Mol Sci. 2020 Jul 28;21(15):5359. doi: 10.3390/ijms21155359 (PMC7432723; doi:10.3390/ijms21155359)
Supplement: Supplementary file 1 [file ijms-21-05359-s001.zip › _IJMS Supplementary materials_proofreading.docx]

**Small extracellular vesicles isolated from serum may serve as signal-enhancers for the monitoring of CNS tumors**

Gabriella Dobra, Matyas Bukva, Zoltan Szabo, Bella Bruszel, Maria Harmati, Edina Gyukity-Sebestyen, Adrienn Jenei, Monika Szucs, Peter Horvath, Tamas Biro, Almos Klekner, Krisztina Buzas

**Supplementary materials**

**Figure S1** **Western blot analyses of classical EV markers.** CD81 as transmembrane protein and Alix as cytosolic protein is showed in all of the four groups to demonstrate the EV nature of isolated nanoparticles.

**Figure S2** **Intragroup Coefficients of variation (CV) distributions.** Histograms demonstrate the CV distributions of the protein MS-signal intensities within each group. The x and y axes show the CV values and the frequency of these values, respectively. Comparing the two sample types, it can be concluded that sEV isolation did not increase variance within groups.

**Figure S3 PCA dotplot constructed after statistical selection based on the means of intensity ratio.** Diagrams visualize the results of the PCA analysis and k-means clustering on whole serum samples (left) and sEV samples (right). X and Y axis of PCA biplots show principal component 1 (PC1) and principal component 2 (PC2) with explained variances. Dots with different forms represent the groups. The colors indicate the clusters formed by k-means clustering. 95% confidence ellipses were constructed around the barycenters of the clusters. Clustering of whole serum samples (left) resulted in a large, inhomogeneous and a single-membered cluster. The homogeneity and completeness scores of the clustering are 0.07 and 0.40, respectively. In the case of EVs (right), k-means clustering formed a homogenous and complete cluster, an inhomogeneous but complete, and two incomplete and inhomogeneous clusters with 0.66 of homogeneity and 0.66 of completeness scores.

Table S1 311 membered protein table of DIA mode constructed spectral library. The proteomic analyses by LC-MS were performed on whole serum samples and sEV samples from patients with GBM, BM, M and CTRL. 4 pools from the 24-24 membered groups were created, allowing four parallel samples to be tested per group. Table contains the measured intensities, ratio, significance and Cohen’s d effect size of the selected proteins. EV markers (based on literature) and proteins of sEV and serum panels are marked. Gene Ontology and Uniprot keyword annotations of each protein are also included.**Table S2** **Sample correlation matrix.** Pearson correlation coefficient matrix Matrix was created based on log_2_(intensity) values of all proteins quantified in each sample. Values below R<0.9 cutoff indicate that the sample has to be excluded from further statistical analysis.

**Table S3** **List of the selected proteins.** Table shows the UniProt ID, Gene symbol, Protein name, ratio of intensity means > 2; < 0.5 and Cohen’s d effect size > 2 parameters of the tumor-ctrl, tumor-tumor comparison-based selected proteins. The two sample groups shared four significantly altered proteins (in bald). These proteins also showed relevant difference in other comparisons, but only the result of the highest Cohen’s d is shown (detailed in Table S1).
