# Supplementary figures and images for: Small Extracellular Vesicles Isolated from Serum May Serve as Signal-Enhancers for the Monitoring of CNS Tumors
Source: Int J Mol Sci. 2020 Jul 28;21(15):5359. doi: 10.3390/ijms21155359 (PMC7432723; doi:10.3390/ijms21155359)

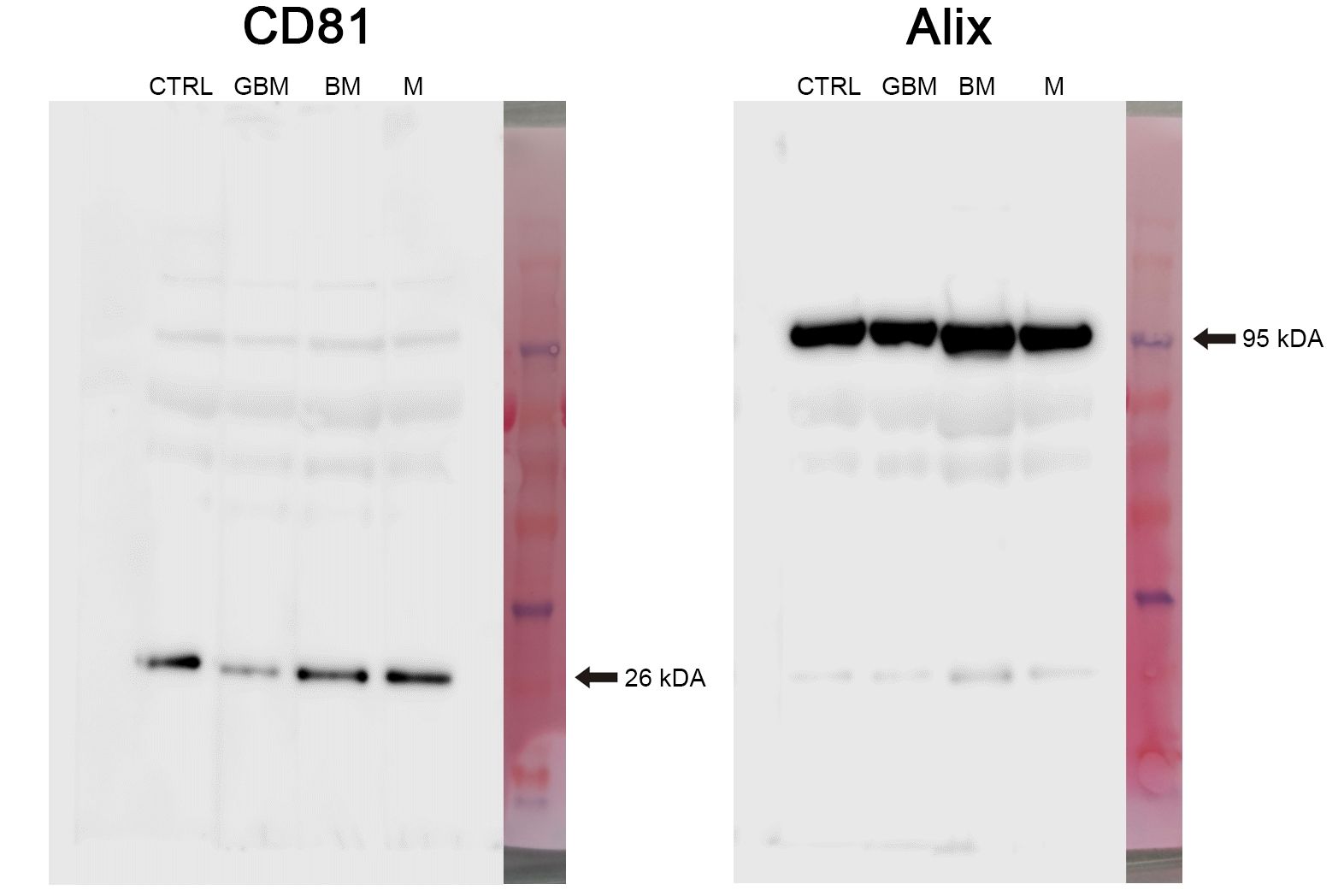

Supplement: Supplementary file 1 [file ijms-21-05359-s001.zip › Figure S1_Western blot analyses of classical EV markers.jpg]

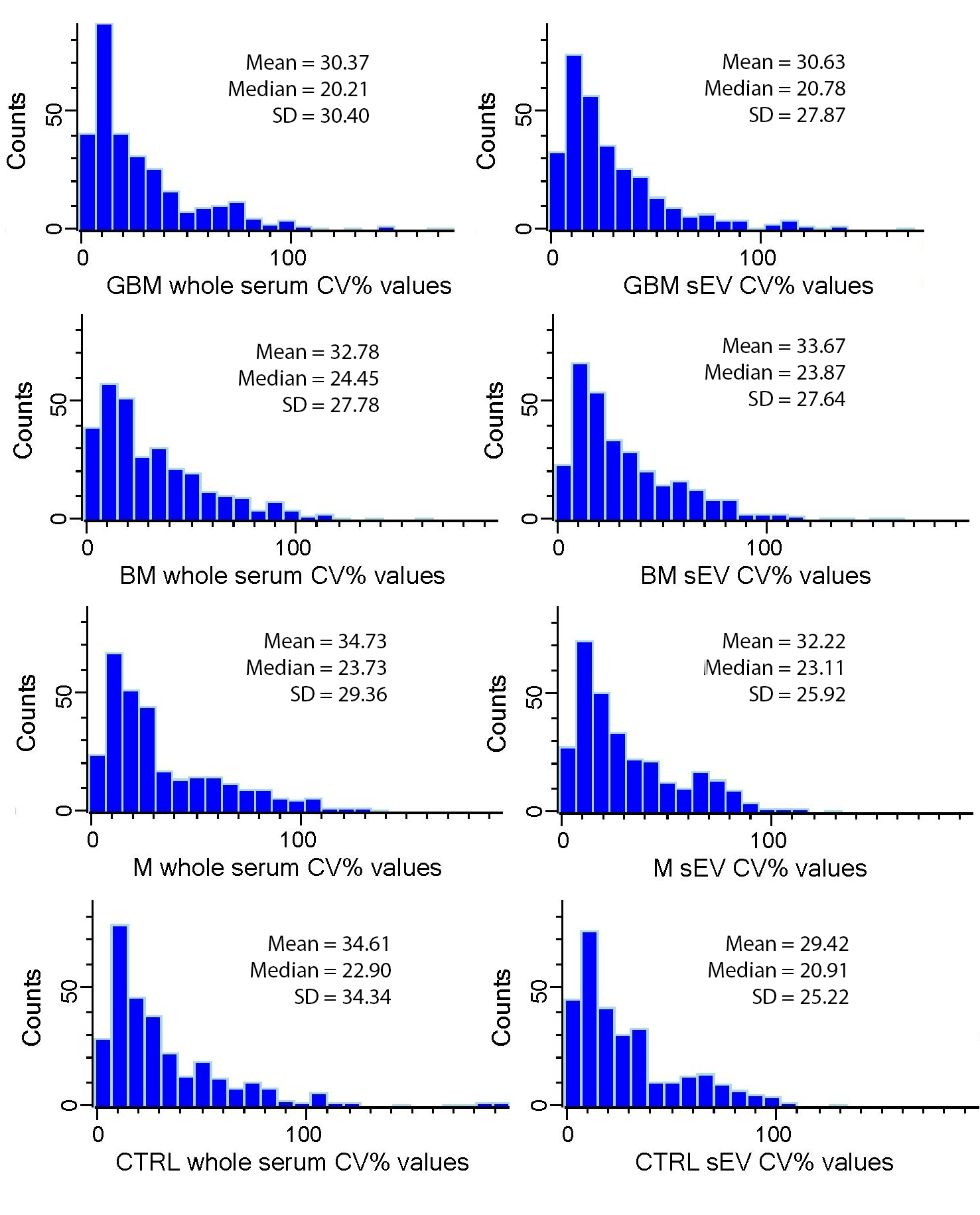

Supplement: Supplementary file 1 [file ijms-21-05359-s001.zip › Figure S2_Intragroup Coefficients of variation (CV) distributions.tif]

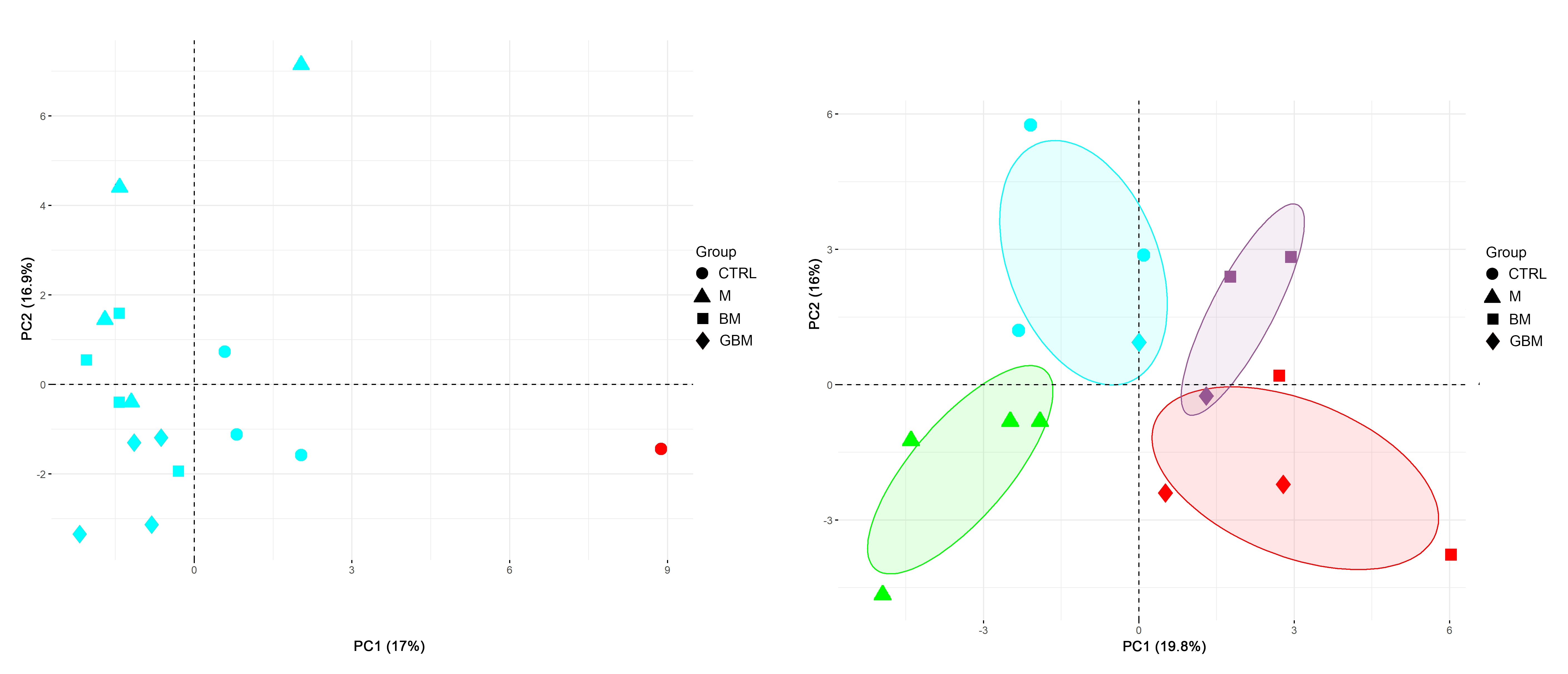

Supplement: Supplementary file 1 [file ijms-21-05359-s001.zip › Figure S3_PCA dotplot constructed after statistical selection based on the means of intensity ratio.tif]
